# The crosstalk between copper-induced oxidative stress and cuproptosis: a novel potential anticancer paradigm

**DOI:** 10.1186/s12964-024-01726-3

**Published:** 2024-07-05

**Authors:** Thi Thuy Tien Vo, Tzu-Yu Peng, Thi Hong Nguyen, Trang Ngoc Huyen Bui, Ching-Shuen Wang, Wei-Ju Lee, Yuh-Lien Chen, Yang-Che Wu, I-Ta Lee

**Affiliations:** 1 Faculty of Dentistry, Nguyen Tat Thanh University, Ho Chi Minh City, 700000 Vietnam; 2 School of Dentistry, College of Oral Medicine, Taipei Medical University, Taipei, 110301 Taiwan; 3 School of Food Safety, College of Nutrition, Taipei Medical University, Taipei, 110301 Taiwan; 4 Department of Anatomy and Cell Biology, College of Medicine, National Taiwan University, Taipei, 100233 Taiwan

**Keywords:** Copper, Cuproptosis, Reactive oxygen species, Oxidative stress

## Abstract

Copper is a crucial trace element that plays a role in various pathophysiological processes in the human body. Copper also acts as a transition metal involved in redox reactions, contributing to the generation of reactive oxygen species (ROS). Under prolonged and increased ROS levels, oxidative stress occurs, which has been implicated in different types of regulated cell death. The recent discovery of cuproptosis, a copper-dependent regulated cell death pathway that is distinct from other known regulated cell death forms, has raised interest to researchers in the field of cancer therapy. Herein, the present work aims to outline the current understanding of cuproptosis, with an emphasis on its anticancer activities through the interplay with copper-induced oxidative stress, thereby providing new ideas for therapeutic approaches targeting modes of cell death in the future.

## Introduction

As copper (Cu) is a trace element in the human body, its intracellular concentration is maintained at a very low level by evolutionarily conserved homeostatic mechanisms that prevent the accumulation of free copper within the cells. It should be noted that copper is a double-edged sword for the cells. Copper is well-recognized as an indispensable cofactor, which plays an important role in the biochemistry of every living organism. However, an overabundance of copper is pernicious to the cells, eventually leading to cell death [1, 2]. The underlying mechanism of copper-induced cell death was unclear until the article “Copper induces cell death by targeting lipoylated TCA cycle proteins” was published by Tsvetkov et al. in 2022. The study sheds light on a novel form of cell death known as cuproptosis, wherein copper binds directly to lipoylated components of the tricarboxylic acid (TCA) cycle, leading to lipoylated protein aggregation and subsequent iron-sulfur cluster protein loss that in turn causes proteotoxic stress and ultimately cell death [3].

The latest estimates of the global burden of cancer highlight a rapidly growing problem, with an estimated 20 million new cases and 9.7 million deaths worldwide in 2022. It is predicted that there will be over 35 million new cancer cases in 2050, an increase of 77% compared to 2022 [4]. Oxidative stress has been demonstrated as one of key mechanisms in the initiation and progression of cancer [5]. The global concept of oxidative stress can be defined as an imbalance between the production of oxidants so-called reactive oxygen species (ROS) and antioxidant defenses in favor of the oxidants, leading to a disruption of redox signaling and control and/or molecular damage [6]. ROS were initially considered extremely harmful and merely associated with tumor-promoting functions. However, it has been found that greater ROS levels that push cancer cells beyond the breaking point lead to the activation of different cell death mechanisms, indicating the tumor-suppressing perspectives [7]. Therefore, the induction of oxidative stress under high levels of ROS can be a potential paradigm in cancer therapy. In the context of this review, it is worth mentioning that copper is a transition metal that can exist in both oxidation and reduction states. Considering the physiological significance of copper as an important cofactor and its unique redox activity, copper homeostasis is essential for a variety of biological processes [1, 8]. Studies have reported high levels of copper in serum and tissues of various types of human cancers such as colorectal cancer [9], liver cancer [10], and lung cancer [11]. Interestingly, there is a growing body of evidence indicating that dysregulation of intracellular copper bioavailability can induce oxidative stress [12–14]. A recent analysis of literature indicates that the global status of copper research not only mainly focuses on the relationship between copper metabolism and oxidative stress but also the anticancer mechanisms of copper [15]. These results suggest that copper, oxidative stress, and cancer are closely connected. Thus, targeting copper may provide a therapeutic advantage for the development of anticancer strategies associated with oxidative stress.

Cuproptosis is a form of copper-dependent regulated cell death, which is novel and distinct from the known cell death mechanisms such as apoptosis, necroptosis, and ferroptosis [3]. Even though the concept of cuproptosis was newly discovered in 2022, pertinent research was performed many years ago. Cuproptosis research dates back to the publication on the toxicity of metal complexes in 1996 [16]. The annual output of this research field has grown steadily ever since. Considering the increasing number of studies indicating the relationship between cuproptosis and cancer process, this cell death mechanism is becoming a hotspot in the research field of cancer therapy [17, 18]. As a result, copper-based interventions that trigger cell death offer a promise for developing novel anticancer strategies. This has raised interest to researchers but lacks scientific analyses that elucidate the underlying mechanisms. Despite extensive research on copper metabolism, oxidative stress, and cuproptosis, existing literature reviews mostly provide overviews of each process separately. There is still a gap in understanding how these mechanisms interact with each other, particularly in the context of cancer. To bridge this gap, the present review aims to provide the current understanding of cuproptosis, with an emphasis on its anticancer activities through the interaction with copper-induced oxidative stress, thereby getting ideas for future research directions in this field. Unveiling the intricate relationship between copper-induced oxidative stress and cuproptosis along with identifying the underlying mechanisms would be beneficial to develop novel and efficient copper-based anticancer strategies.

## The relationship between copper metabolism and oxidative stress

**Fig. 1:**
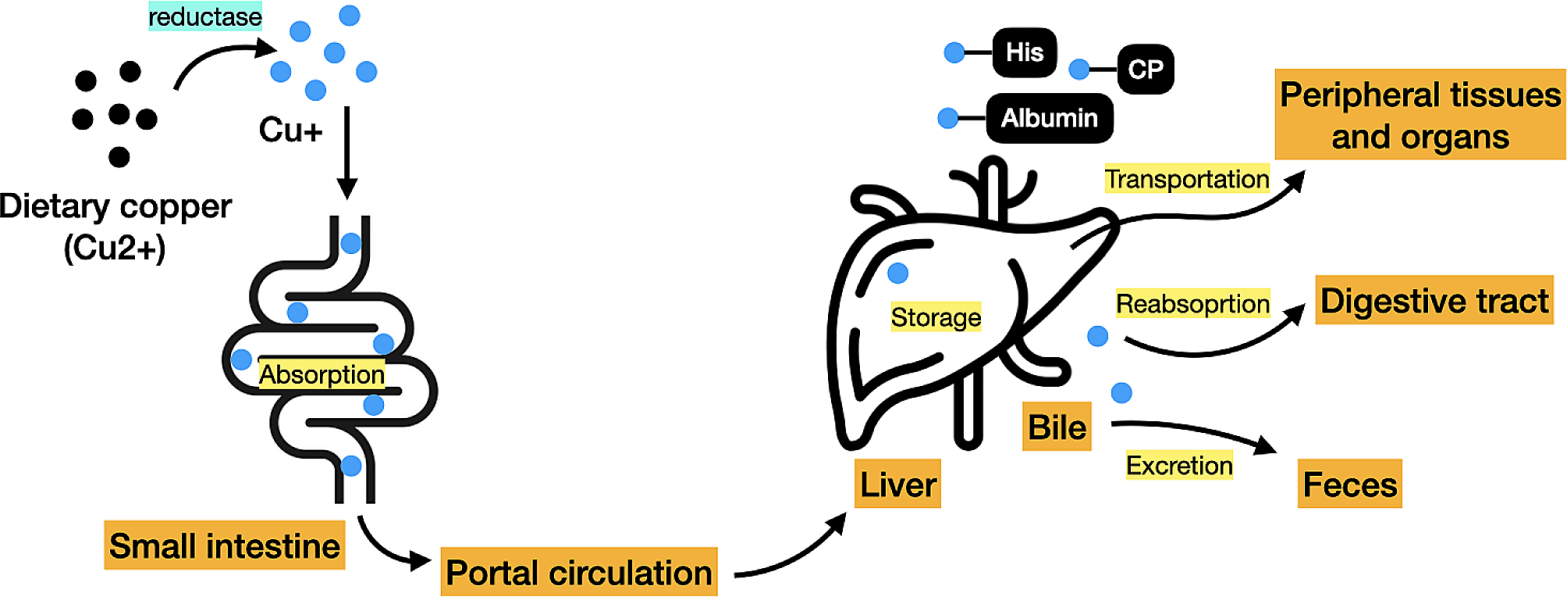
Schematic illustration of copper metabolism. Dietary copper, mostly exists in the Cu2 + form, is converted to Cu + through reductase actions, which is primarily absorbed by the small intestine and in turn directed to the liver for storage and transportation through the portal circulation. The copper is further distributed to other peripheral tissues and organs via the bloodstream. Serum copper are bound to proteins such as ceruloplasmin (CP), albumins and free amino acids instead of being free. Excess copper is exported to the bile, which is either excreted through feces or reabsorbed through the digestive tract

Since both insufficient and superfluous copper levels are detrimental to the cells, copper homeostasis must be maintained in living organisms. Otherwise, a copper dyshomeostasis may contribute to a plethora of pathological conditions. Being an important trace element in the human body, the metabolism of copper is a complex process (Fig. 1). Copper is found in organisms in two different states, an oxidized state Cu(II)/Cu2 + and a reduced state Cu(I)/Cu+. Copper is primarily obtained from the diet, which mostly exists in the Cu2 + form [1]. The extracellular copper in the form of Cu2 + is directly transported by divalent metal transporter 1 (DMT1), or also called solute carrier family 11 member 2 (SLC11A2), but unable to be used directly by the cells [19, 20]. Alternatively, copper is typically converted from Cu2 + to Cu + through metalloreductases and combines with copper transporter 1 (CTR1), or also called solute carrier family 31 member 1 (SLC31A1), for absorption [21, 22]. Copper uptake chiefly occurs in the small intestine, from where it is pumped into the portal circulation, and subsequently reaches the liver, which is the principal organ in the body for copper storage and excretion. In the liver, copper is further released into the circulation in order to reach other tissues and organs. Copper ions are transported in the blood by binding to proteins instead of being free, which can be absorbed by CTR1 upon arrival at peripheral tissues and organs. Most of serum copper are bound to ceruloplasmin (CP) while the remaining are bound to albumins and free amino acids. Excess copper is exported to the bile, most of which is excreted through feces whereas a small part is reabsorbed through the digestive tract [22, 23].

**Fig. 2:**
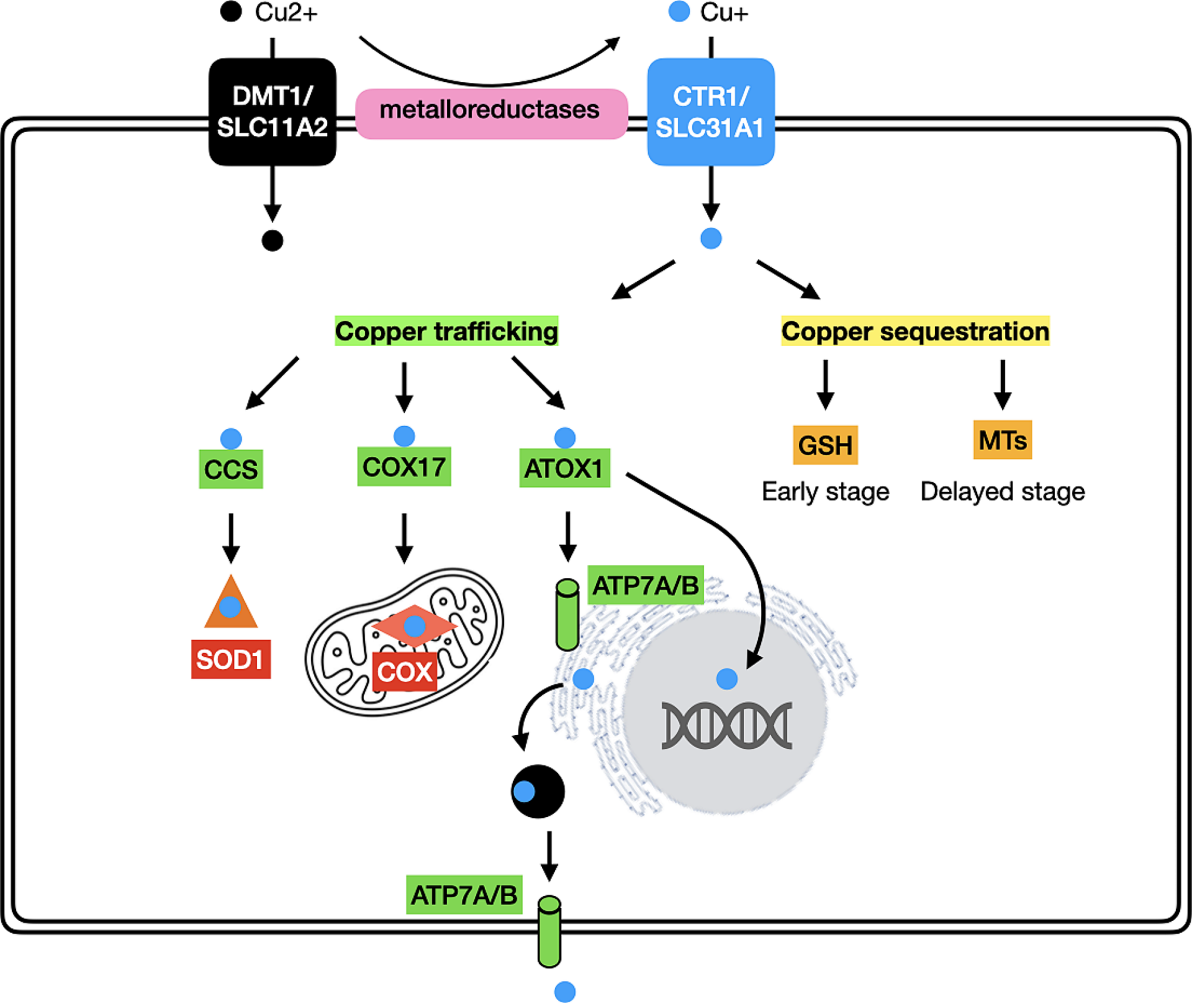
Schematic illustration of intracellular copper trafficking and sequestration. Since the extracellular copper in the form of Cu2 + is directly transported by divalent metal transporter 1 (DMT1), or also called solute carrier family 11 member 2 (SLC11A2), but unable to be used directly by the cells, it is typically converted to Cu + through metalloreductases. The entry of Cu + is mainly controlled in a manner dependent on copper transporter 1 (CTR1), or also called solute carrier family 31 member 1 (SLC31A1). After incorporation, the ions are delivered to various cellular components including the cytoplasm, mitochondria, Golgi apparatus, and nucleus by a sophisticated system of high-affinity copper chaperones. In the cytoplasm, copper chaperone for superoxide dismutase (CCS) transports Cu + to superoxide dismutase (SOD1). Cu + is also directed to the mitochondria by cytochrome c oxidase copper chaperone 17 (COX17), where it is used by cytochrome c oxidase (COX) to activate the activity of enzymes in the respiratory chain. Another important copper chaperone is antioxidant 1 copper chaperone (ATOX1), which delivers Cu + to the nucleus. ATOX1 also transports Cu + to ATPase copper-transporting α (ATP7A) and ATPase copper-transporting β (ATP7B) located in the trans-Golgi network, both of which play a role for copper distribution and copper excretion. The sequestration of excess Cu + is determined by molecules such as GSH and metallothioneins (MTs)

The copper homeostasis should be remained steady at the cellular level. The intracellular copper trafficking is tightly regulated by a sophisticated system of high-affinity copper chaperones, which deliver copper to various cellular components including the cytoplasm, mitochondria, Golgi apparatus, and nucleus for further cellular metabolic processes (Fig. 2). In the cytoplasm, copper chaperone for superoxide dismutase (CCS) transports copper to superoxide dismutase (SOD1), which exerts an antioxidative role [24]. Copper is also directed to the mitochondria, where it is used by cytochrome c oxidase (COX) to activate the activity of enzymes in the respiratory chain. Cytochrome c oxidase copper chaperone 17 (COX17), which shuttles between the mitochondria and the cytoplasm, is in charge of carrying copper from the cytosol to the mitochondrial intermembrane space [25]. Another important copper chaperone is antioxidant 1 copper chaperone (ATOX1), which delivers copper to the nucleus for driving gene expression. In addition, ATOX1 transports copper to ATPase copper-transporting α (ATP7A) and ATPase copper-transporting β (ATP7B). Under physiological condition with low copper levels, these copper-transporting ATPases are located in the trans-Golgi network (TGN), pumping copper from the cytoplasm into the TGN lumen. In response to intracellular copper increases, they translocate and fuse with the plasma membrane to release the ions into the extracellular environment, which are recycled back to the TGN upon copper levels returning to normal [26]. It should be noted that copper-transporting ATPases are expressed in a tissue-specific manner. ATP7A is found in nearly every cell in the body, except for hepatocytes, where it is replaced by the paralogue ATP7B. In enterocytes, ATP7A carries copper from the Golgi apparatus to the basolateral region of the cells, releasing the ions into the portal circulation and eventually reaching the liver for uptake by hepatocytes in a CTR1-dependent manner. Under normal copper levels, hepatic ATP7B located in the TGN transports the ions to CP for copper distribution to peripheral tissues and organs via the bloodstream. However, during elevated copper levels, hepatic ATP7B translocates to the lysosome to activate exocytosis, leading to the biliary excretion of excess copper [20]. It is worth mentioning that excess copper within the cells must be sequestrated since free copper ions are capable of producing intracellular ROS. Metallothioneins (MTs), with thiol groups in cysteins, is known as an efficient chelator of a significant amount of copper ions that enter the cells via CTR1, preventing cytotoxicity. However, the cells are still susceptible to copper toxicity because the protective effects of MTs require hours to exert upon exposure. It is proposed that glutathione (GSH), a tripeptide consisting of glutamate, cysteine, and glycine residues, serves as a key element that chelates and detoxifies copper ions just as they enter the cells [27].

The relationship between copper metabolism and cancer pathogenesis has been extensively investigated. A number of observations have indicated elevated copper concentrations in serum or tissues of patients with different types of cancers such as colorectal cancer [9], liver cancer [10] and lung cancer [11]. Importantly, many studies have demonstrated a significant decline in serum copper levels in patients with various malignant diseases undergoing cancer therapy [28–30]. A transcriptome analysis of copper homeostasis-related genes found a strong elevation in mRNA levels of CTR1 protein encoded by SLC31A1 gene, which was accompanied by an increase in transcript levels for ATP7A, copper metabolism Murr1 domain containing 1, the COX assembly factors, the cupric reductase six transmembrane epithelial antigen of the prostate, and the metal-regulatory transcription factors and specificity protein 1 in colorectal carcinoma samples [31]. These findings suggested a demand for higher levels of copper in cancer cells to meet their energy requirement for rapid proliferation compared to normal cells. This may be due to the fact that copper acts as a cofactor for COX in the mitochondria, which plays a crucial role in the electron transport chain (ETC) and ATP synthesis in the mitochondrial respiratory chain. Copper is also a cofactor of ferroxidases, thereby regulating the mitochondrial uptake of iron that is a critical element in not only iron-sulfur cluster protein assembly and heme biosynthesis but also metabolic balance and function within mitochondria [32]. It is known that many tumors can produce elevated levels of lactate even in the presence of oxygen, reflecting aerobic glycolysis or “Warburg effect”, whereas normal tissues typically use mitochondrial respiration. However, alleviating systemic copper with a chelating drug can compromise mitochondrial energy metabolism and attenuate ATP levels despite induction of glycolysis [33]. Therefore, increased copper levels may act as a limiting nutrient in tumorigenesis through metabolic reprogramming that can switch the energy-producing pathways of cells between glycolysis and oxidative phosphorylation (OXPHOS), determining cell fate. In addition, literature has documented that copper can affect multiple molecules involved in various signaling pathways to activate related cascades, directly or indirectly contributing to tumor progression such as tumor growth, angiogenesis, and metastasis. It was demonstrated that higher serum copper concentrations might be associated to the progression of non-alcoholic fatty liver disease-cirrhosis toward hepatocellular carcinoma. The study also highlighted that high extracellular copper concentrations promoted cell growth, migration, and invasion of liver cancer cells by upregulating MYC oncogene and modulating MYC/CTR1 axis. In a model of mouse embryonic fibroblasts, the ATOX 1 copper chaperone exhibited a novel role of a copper-dependent transcription factor involved in cell proliferation with a significant increase in cyclin D1 expression and entry into S phase [34]. Another investigation using mouse hindlimb ischemia model further indicated the involvement of ATOX1 in inflammatory neovascularization via chaperone and transcription factor function [35]. The angiogenic effect of copper has been well-recognized, which is presumed to attribute to the regulation of factors involved in vessel formation and maturation, especially the pro-angiogenic factor, namely vascular endothelial growth factor (VEGF), and its major transcription factor, namely hypoxia-inducible factor-1-alpha (HIF1α) [36]. Copper not only promotes the proliferation and angiogenesis of tumor cells but also their metastasis. The copper-transporting ATP7A is required for the delivery of copper to copper-dependent metalloenzymes called lysyl oxidases (LOX) that play a crucial role in metastatic mechanism. In mouse models of breast cancer and lung cancer in situ, deletion of ATP7A inhibited LOX activity, leading to a significant loss of tumor growth and reduction of tumor metastasis [37]. Considering the multifaceted role of copper in oncology, a thorough understanding of underlying mechanisms is required to harness the interplay between copper homeostasis with cancer initiation and progression for improving treatment outcomes in cancer patients.

Every cell within the human body strives to sustain a state of equilibrium between the oxidant and antioxidant species, both of which are crucial for cellular metabolism, signal transduction and biological functions. ROS, commonly known as highly reactive oxygen-containing free radicals and non-radical intermediates, are inevitable by-products of aerobic metabolism [38]. The vast majority of intracellular ROS is continuously generated from mitochondria at the ETC, most of which is reduction of molecular oxygen, resulting in superoxide anion (O_2_^•^−). This short half-life species can further generate hydrogen peroxide (H_2_O_2_) through either spontaneous or enzymatic dismutase reactions and in turn hydroxyl radicals (OH^•^) via Fenton and Haber-Weiss reactions catalyzed by transition metal ions. Under physiological conditions, the cells rely on scavenging systems such as GSH, SOD1, and other antioxidant defense components to sequester intracellular ROS, thereby maintaining the redox homeostasis [39]. When the levels of intracellular ROS surpass the counteracting capacity of intrinsic antioxidant defenses, oxidative stress arises and causes oxidative damage to biological molecules, leading to signaling disruption and eventually cell death [6]. In the human body, copper exists in both oxidation states, acting as either a recipient or a donor of electrons. This unique characteristic endows copper with the ability to serve as a vital cofactor in redox reactions of various essential enzymes, especially SOD1 involved in ROS detoxification and COX involved in the mitochondrial ETC as mentioned above. Thus, copper is indispensable for cellular metabolism, particularly the mitochondrial respiratory chain [20, 23]. A growing number of evidence has demonstrated the ability of chronic copper overload and/or excess copper accumulation to initiate oxidative damage and compromise cellular events [40–42]. It is presumed that the basis for underlying mechanisms is the propensity of both Cu2 + and Cu + ions to participate in redox reactions. During the switch between Cu + and Cu2 + states, electron transfer leads to the production of ROS, including O_2_^•^−, H_2_O_2_, and OH^•^, via Fenton reactions. Moreover, Cu2 + can be reduced to Cu + in the presence of O_2_^•^−, which is capable to catalyze the conversion of OH^•^ from H_2_O_2_ through Haber-Weiss reactions [13]. This results in the transition of copper ions between oxidized and reduced states, generating OH^•^, which are considered the most powerful oxidizing radicals in biological systems and extremely reactive with biological molecules, at a substantial rate [38]. Therefore, surplus copper has been recognized as a potent oxidant that increases ROS generation and promotes oxidative stress. This even creates a vicious cycle where high ROS levels further damage mitochondria, leading to more ROS production. In the context of cancer, oxidative stress can induce DNA damage and mutations, resulting in genomic instability, the activation of oncogenes, and the inactivation of tumor suppressor genes [5]. Mechanistically, ROS can activate numerous signaling pathways that contribute to cancer initiation and progression such as, but not limited to, the mitogen-activated protein (MAP) kinases (MAPKs), the phosphatidylinositol 3-kinase (PI3K)/protein kinase B (AKT), and the Janus kinase (JAK)/signal transducer and activator of transcription (STAT) pathways [43]. ROS can activate MAPKs, leading to the phosphorylation and activation of downstream targets, for instance, AP-1 and NF-*κ*B transcription factors, which in turn regulate the expression of genes involved in cell survival, proliferation, and apoptosis [44]. The PI3K/AKT/mammalian target of rapamycin (mTOR) pathway, another crucial signaling pathway modulated by ROS, is known to play a key role in numerous cellular functions, including proliferation, adhesion, migration, invasion, metabolism, and survival. Moreover, ROS-dependent angiogenesis can be induced through the activation of this pathway to lead to the expression of HIF1α and VEGF [45]. The modulation of JAK/STAT pathway is also susceptible to ROS. Oxidative stress can induce the activation of the JAK/STAT pathway, which in turn regulates the expression of genes involved in inflammation, cell proliferation, and apoptosis [46]. Interestingly, copper as a catalytic cofactor in redox chemistry can modulate the activity of not only various metabolism-related enzymes – e.g., COX, SOD1, ferroxidase, and LOX as mentioned above, but also some kinases. The first evidence about the role of copper as a positive allosteric regulator of kinases activity dates back to 2012. Mitogen-activated protein kinase kinase 1/2 (MEK1/2) was the first-identified copper-binding kinase. Depletion of Ctr1 high-affinity copper transporter, mutation of Ctr1, and utilization of copper chelators all inhibited the ability of the MAP kinase kinase Mek1 to phosphorylate the MAP kinase Erk in vitro. Subsequent experiments revealed that recombinant Mek1 can bind two copper atoms with high affinity, promoting Mek1 phosphorylation of Erk in a dose-dependent manner. Consistently, knockout of Ctr1 also led to a significant reduction in Erk phosphorylation in cardiac tissues in mice [47]. Further studies on the ability of copper as a dynamic signal metal of the MEK axis have demonstrated the involvement of other copper chaperones, including CCS [48] and ATOX1 [49]. These findings supported the role of copper in the activation of MEK/ERK pathway by binding to allosteric sites, whose dysregulation is closely related to cancer initiation and progression. In addition to MEK/ERK signaling cascade, copper was demonstrated to induce the PI3K/AKT signaling pathway by binding 3-phosphoinositide dependent protein kinase 1 to activate its downstream AKT kinase in a CTR1-dependent manner, contributing to tumorigenesis [50]. Similarly, copper was shown to enhance the enzymatic activity of casein kinase 2 (CK2) both in vitro and in vivo, suggesting CK2 as a copper-regulated kinase [51]. Among other functions, CK2 plays a vital role in many oncogenic signaling pathways such as the PI3K/AKT [52], IKK (IκB) kinase/NF-κB [53], and JAK/STAT pathways [54]. Taken together, copper and ROS can synergistically regulate different signaling pathways associated to tumorigenesis and tumor progression. This underscores the importance of maintaining copper homeostasis and redox balance to prevent cancer initiation and development.

## Cuproptosis: the bright side of copper overload in cancer

### A snapshot on cuproptosis mechanism

**Fig. 3:**
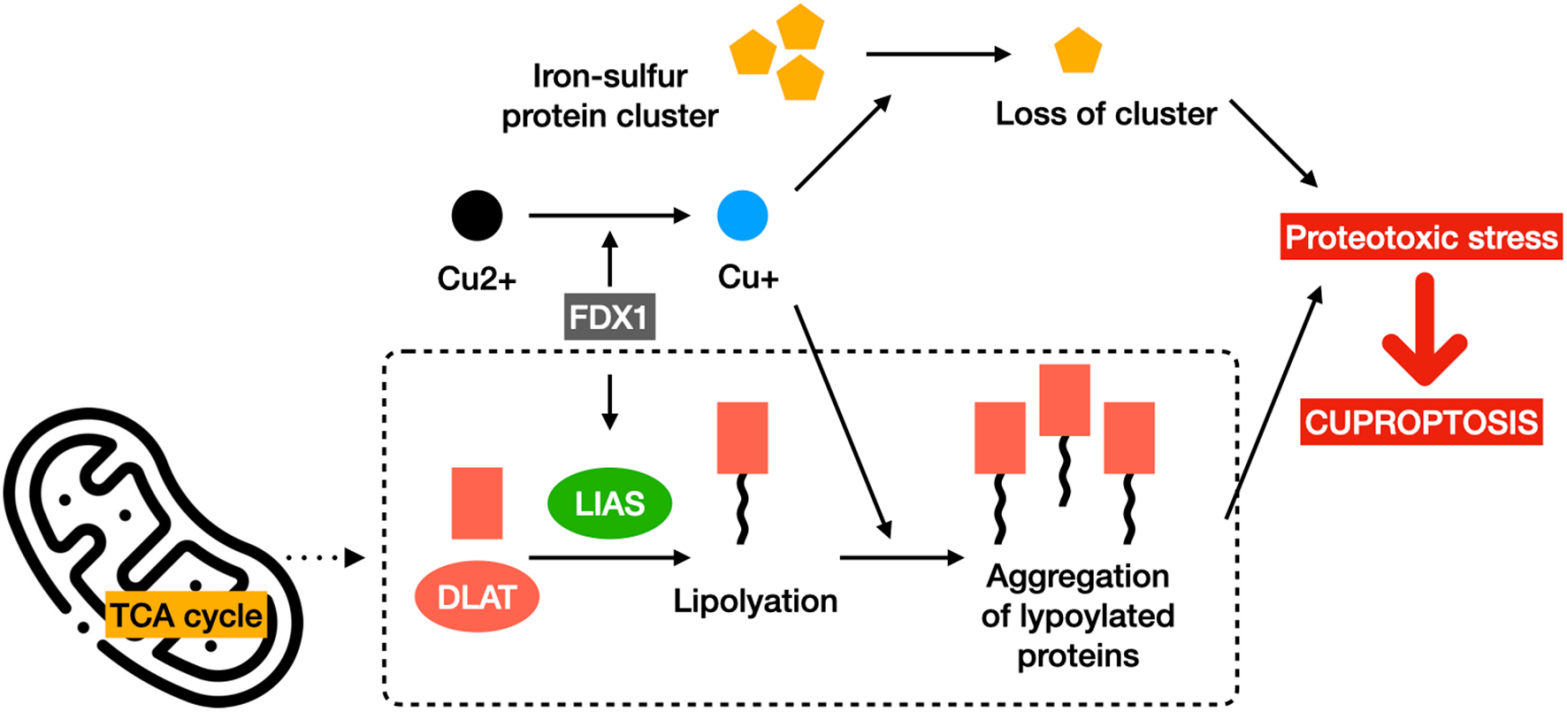
Schematic illustration of cuproptosis mechanism. Cuproptosis is a copper-dependent form of regulated cell death that is distinct from other known mechanisms including apoptosis, necroptosis, and ferroptosis. Mechanistically, ferrodoxin 1 (FDX1) encodes a reductase to reduce Cu2 + to Cu + and promotes the lipoylation of dihydrolipoamide S-acetyltransferase (DLAT), a protein target involved in the TCA cycle. Excess Cu + leads to the loss of iron-sulfur protein clusters and aggregation of lipoylated proteins, triggering proteotoxic stress and eventually cell death

Across the animal kingdom, from prokaryotes to eukaryotes, copper homeostasis is finely and intricately regulated to prevent the cells from copper overload that poses a threat to cell survival. While many forms of cell death such as apoptosis, necroptosis, pyroptosis, and ferroptosis have been extensively investigated, the precise mechanism by which copper ions induce cellular toxicity and cell death remains unclear [55]. In 2022, Tsvetkov et al. indicated that copper toxicity was significantly linked to mitochondrial activity, and copper accumulation led to cell death through the involvement of crucial components of the TCA cycle. The study for the first time proposed the concept of cuproptosis as a unique cell death pathway that is distinct from all other known ones. In cuproptosis, copper binds to the lipoylated proteins of the TCA cycle, resulting in lipoylated protein aggregation and iron-sulfur cluster loss that lead to proteotoxic stress and ultimately cell death (Fig. 3) [3]. The main findings of this pioneer study regarding cuproptosis mechanism are summarized as below.

First, copper ionophores are small molecules that bind and shuttle copper into the cells, which are valuable tools to elucidate the mechanisms of copper toxicity [56]. Tsvetkov et al. observed that the copper ionophore called elesclomol (ES) caused cell death mainly dependent upon copper overload. The data indicated that ES-induced cell death did not involve either the cleavage or activation of caspase 3 activity, known as a hallmark of apoptosis. Moreover, neither deletion of the crucial effectors of apoptosis, namely BAX and BAK1, nor co-treatment the cells with pan-caspase inhibitors impacted on ES-induced cell death, suggesting that copper-induced cell death differed from apoptosis. Similarly, pharmacological treatment with inhibitors of either necroptosis or ferroptosis also had no effect on ES-induced cell death [3]. These results propose that the mechanism of cuprotosis is distinct from that of other known forms of cell death.

Second, Tsetkov et al. further established the association between copper-induced cell death and mitochondrial metabolism. The authors found that mitochondrial respiration-reliant cells were significantly more susceptible to ES than glycolysis-reliant ones, indicating a substantial impact of mitochondrial function on the sensitivity to copper ionophores. Moreover, treatment with inhibitors targeting the ETC and mitochondrial pyruvate uptake markedly reduced cell death sensitivity but did not the mitochondrial uncoupling agent, suggesting that copper-induced cell death mainly linked to mitochondrial respiration rather than ATP production. Cell culture under hypoxic conditions also led to a considerable decrease in cell death sensitivity as compared to under normoxic conditions, emphasizing the role of cellular respiration in copper-induced cell death. However, ES was shown to significantly attenuate the respiratory reserve capacity rather than the rate of basal or ATP-related respiration, demonstrating that copper did not target the ETC directly but the TCA cycle components [3]. Therefore, further experiments are required to explore the precise mechanisms underlying the relationship between copper-induced cell death and the TCA cycle.

Third, lipoylation is a highly conserved posttranslational modification in which a lipoamide is covalently attached to a lysine residue through an amide bond. In mammals, this phenomenon is recognized to merely occur on four essential multimeric metabolic complexes that regulate specific carbon entry points into the central metabolic pathway of the TCA cycle [57]. Tsetkov et al. identified the specific metabolic pathways involved in copper toxicity based on a remarkable reduction in cell death induced by copper ionophores when silencing seven genes. These genes included one encoding ferredoxin 1 (FDX1), those encoding the components of the lipoic acid pathway, namely lipoyltransferase1 (LIPT1) and lipoic acid synthetase (LIAS), and those encoding the protein targets of lipoylation in the pyruvate dehydrogenase complex, namely dihydrolipoamide dehydrogenase (DLD), dihydrolipoamide S-acetyltransferase (DLAT), pyruvate dehydrogenase alpha 1 (PDHA1), and pyruvate dehydrogenase beta (PDHB). FDX1, an iron-sulfur protein that can reduce Cu2 + into its more toxic form Cu1 + and is also a direct target of ES, was proved to be an upstream regulator of protein lipolylation. Additionally, in FDX1 knockout experiments, there was a significant decrease in protein lipoylation of DLAT and another TCA cycle component called dihydrolipoamide S-succinyltransferase (DLST), both of which no longer bound copper, suggesting the necessity of lipoyl moiety for copper binding. Besides, a model was proposed, where the toxic gain of function of lipoylated proteins, specifically DLAT, following exposure to copper ionophores was mediated by their abnormal oligomerization. Mass spectrometric analysis showed that copper ionophore treatment led to iron-sulfur cluster protein loss and subsequent proteotoxic stress in an FDX1-dependent manner, further emphasizing the interaction between FDX1, protein lipoylation machinery, and copper toxicity [3].

Finally, Tsetkov et al. reported that copper-induced cell death shared the same mechanisms across different genetic models of copper homeostasis dysregulation. A cellular model that overexpressed the SLC31A1 protein exhibited a greater cellular sensitivity to copper ions at physiological concentrations. In order to verify the in vivo mechanisms, an aged Atpb–/– animal model was investigated. The results showed that the deletion of this copper transporter markedly enhanced intracellular copper accumulation. Moreover, the Atp7b−/− mice had significantly lower levels of iron-sulfur cluster and lipoylated proteins compared to both Atp7b−/+ and wild-type control mice [3].

Taken together, this groundbreaking study provides valuable insights into the mechanisms underlying cuproptosis. The findings not only support the notion that mitochondria represent a multifaceted regulator of cell death, including copper-induced cell death, but also reinforce the concept that oxidative stress is a fundamental mechanism of metal-induced toxicity. Despite these major advances, further research is required to fully explore the complexities of this novel cell death form for developing potential therapeutic interventions and combination treatments.

### Cuproptosis: a novel cell death mechanism in cancer

Cuproptosis is a newly discovered form of programmed cell death dependent on copper bioavailability. Investigating in vitro and in vivo models of intervertebral disc degeneration revealed the relationship between oxidative stress and cuproptosis, in which oxidative stress increased copper influx by enhancing the expression of CTR1 and ATP7A and subsequently promoted cuproptosis in the presence of copper by upregulating FDX1 and TCA-related proteins [58]. This study highlighted the importance of oxidative stress as an intermediate node linking copper overload and cuproptosis induction. Similarly, quantitative proteomics revealed the crosstalk between copper stress and cuproptosis in cancer cells. It was indicated that copper stress not only led to ROS overproduction, oxidative damage and cell cycle arrest but also induced cuproptosis. Upon copper treatment, positive cuproptosis mediators were down-regulated, whereas negative those were up-regulated, proposing a feedback protection mechanism of cuproptosis in cancer to against excess copper uptake. Nevertheless, adequate copper intake inevitably resulted in cancer cell death by cuproptosis [59]. Cuproptosis, therefore, has a high potential for clinical translation in cancer therapy. However, the exact mechanisms by which cuproptosis operates in cancer cells remain unclear, and a large number of studies are needed to unveil the relationship between cuproptosis and cancer process. As research continues, it is believed that novel targets would emerge to provide ideas for therapeutic strategies and bring new hope for cancer patients.

Since copper ions are primarily stored in mitochondria, any imbalance in intracellular copper metabolism can exert cytotoxic effects and lead to pathological processes. Cuproptosis is considered as a copper-triggered modality of mitochondrial cell death, which may be related to altered mitochondrial activity [60]. A recent work exploiting the antitumor activity and mechanism of the Cu(I)-based mitochondria-targeting therapy reported that a mitochondria-targeting Cu(I) complex, namely Cu(I)Br(PPh3)3 (CBP), exhibited antitumor and antimetastatic efficacy both in vitro and in vivo by specifically targeting mitochondria and inducing mitochondrial dysfunction. CBP significantly induced intracellular ROS generation through a Fenton-like reaction. Additionally, the complex instigated the oligomerization of lipoylated proteins and the loss of Fe-S cluster proteins, consistent with characteristics of cuproptosis. The cytotoxicity of CBP was only reversed by a copper chelator rather than inhibitors of the known cell death, further indicating copper-dependent cytotoxicity [61]. Moreover, cuproptosis is known to rely on mitochondrial respiration, in which OXPHOS is an indispensable constituent. A study employing the OXPHOS and cuproptosis gene set scores reported a correlation between high cuproptosis-OXPHOS status with high immunosuppressive signal and poor prognosis in esophageal squamous cell carcinoma. Furthermore, the construction of a regulatory network in the high cuproptosis-OXPHOS group identified high-mobility group A1 (HMGA1) as a critical transcription factor that modulates cuproptosis, offering new insights into targeted therapeutic strategies [62].

Cuproptosis-relevant genes (CRGs) can be divided into two groups, one is the lipoic acid pathway – i.e., FDX1, LIAS, LIPT1, DLD, and the other is pyruvate dehydrogenase complex – i.e., DLAT, PDHA1, PDHB, metal-regulatory transcription factor-1 (MTF1), glutaminase (GLS), and cyclin-dependent kinase inhibitor 2 A (CDKN2A) [3]. One of indicators of cancer is the disturbance of cellular metabolism [63]. Through regulation of cell metabolism, CRGs may modulate the onset and progression of cancer. A case-control cohort showed that FDX1 was associated with risk of lung cancer [64]. An analysis of CRGs demonstrated the prognostic value of these genes, especially LIPT1, in melanoma and the correlation between LIPT1 expression with immune infiltration [65]. Similarly, a series of bioinformatics analyses and experimental validation elucidated the potential role and possible mechanisms of cuproptosis in gynecological tumors. The expression and DNA alteration profiles of CRGs were also reported. These results suggested that cuproptosis was closely related to gynecological oncology and attributed to metabolism dysregulation. Moreover, FDX1 could affect CD8 + cell infiltration in gynecological cancer patients, implicating the effect of cuproptosis on immune infiltration [66]. In addition, cell death can be recognized as a pathological event associated with tissue damage and sterile inflammation [67]. However, the inflammatory response to cuproptotic death remains unknown. It was reported that high-mobility group box 1 (HMGB1), a damage-associated molecular pattern, was released during cuproptosis to initiate inflammation. Mechanistically, copper accumulation can induce ATP depletion, which in turn activated AMP-activated protein kinase to promote HMGB1 phosphorylation and enhance its release. Functionally, HMGB1 was demonstrated as a mediator of cuproptotic cell-induced macrophage activation [68]. These findings provided new insights into our understanding of cuproptosis-related inflammatory responses. The relationship between cuproptosis and cancer immunity has yet to be fully explored, indicating a direction worth investigating in the future.

### The crosstalk between copper-induced oxidative stress and cuproptosis in cancer therapy

Cancer is a life-threatening disease, posing a serious health burden in the world. Along with a continuous research progression over years, it is well-acknowledged that induction of cancer cell death is a viable approach for cancer treatment. Cell death is broadly categorized into accidental cell death and regulated cell death [69]. The latter is commonly defined as a spontaneous, programmed cell death finely determined by effector molecules and strictly regulated by signaling pathways, which can be further divided into apoptosis (1972), lysosomal cell death (2000), pyroptosis (2001), netotic cell death (2004), necroptosis (2005), immunogenic cell death (2005), entotic cell death (2007), parthanatos (2009), ferroptosis (2012), autophagic cell death (2013), alkaliptosis (2018), and oxeiptosis (2018) [70]. Different types of regulated cell death have been extensively investigated in the field of cancer therapy, providing valuable insights for the development of effective therapeutic strategies [71–73]. However, the acquisition of resistance to cell death is one of hallmarks of cancer cells that impedes the efficacy of conventional treatment, remaining a major challenge to overcome [74]. Therefore, it is crucial to decipher the underlying mechanisms of cell death pathways and reveal the complex interactions between molecules involved in cell survival and death, thereby identifying specific regulatory molecules and offering novel targets for targeted cancer therapy. This would help to maximize the selective elimination of cancer cells while preserving normal cells, enabling precise treatment of cancer.

The linkage between cancer and oxidative stress has been the subject of intense debate. In normal cells, a delicate equilibrium exists between ROS generation and antioxidant defenses. However, in cancer cells, the rapid cell growth demands much more energy from the mitochondria, promoting ROS generation. The failure of cellular antioxidants to counteract excess ROS eventually results in oxidative stress. Consequently, cancer cells undergo oxidative stress due to continuous overproduction of intracellular ROS compared to normal cells. While oxidative damage can be efficiently fixed with the aid of specialized repair system, irreversible damage to biomolecules and lipid peroxidation due to the lack of cellular repair processes can result in structural and functional changes that ultimately cause cell rupture [5, 7]. The direct evidence of oxidative stress in cancer cells is demonstrated by the significantly elevated levels of ROS in cancer cells compared to normal cells [75, 76], while the indirect proof is obtained through the markedly increased levels of lipid peroxidation products [77, 78]. Many studies have also indicated the presence of oxidative stress in various types of human tumors compared to normal tissues [78–80], along with the alterations in the levels of antioxidant enzymes such as SOD in tumor tissues [81, 82]. Oxidative stress is well-known to closely associate with the initiation and progression of cancer. Nonetheless, cancer cells under sustained and increased levels of ROS may overwhelm ROS detoxifying capacity and fail to adapt to further oxidative insults, eventually triggering cell death [5, 7]. Literature has indicated that different types of cell death share ROS as the same effector molecules, highlighting an interplay between signaling pathways. It has been long documented that ROS can induce apoptosis in both extrinsic and intrinsic pathways [83]. A growing number of publications has well documented that ROS also play a central role in various non-apoptotic forms of regulated cell death, including lysosomal cell death [84], pyroptosis [85], netotic cell death [86], necroptosis [87], parthanatos [88], ferroptosis [89], autophagic cell death [90], and oxeiptosis [91]. Considering the cell versatility in switching between different forms of cell death that share ROS signaling pathways, it has been proposed to exploit the vulnerability of cancer cells to oxidative stress as a potential therapeutic strategy for cancer treatment.

**Fig. 4:**
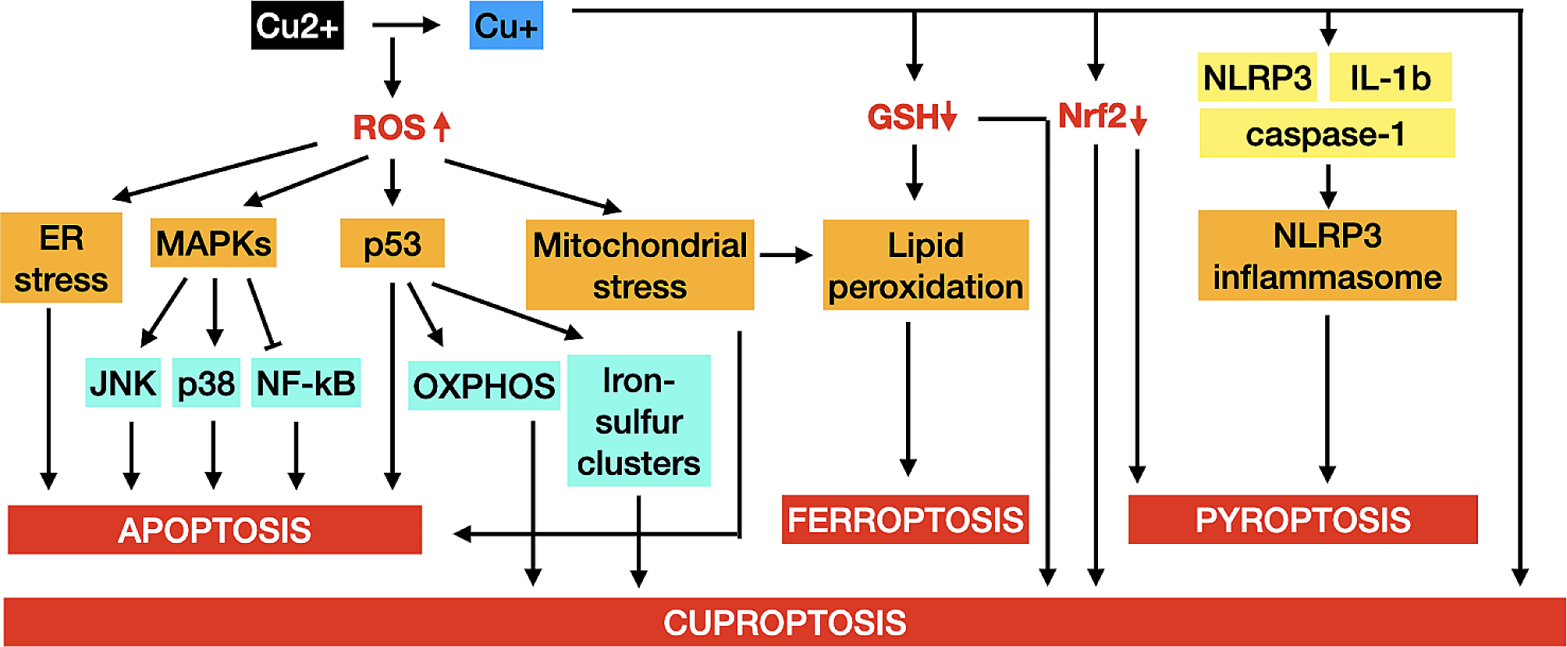
Schematic illustration of the crosstalk between cuproptosis and other regulated cell death pathways. Cuproptosis is a unique form of regulated cell death that is dependent on copper accumulation. Moreover, copper can induce or catalyze the breakdown of hydrogen peroxide into hydroxyl radicals via Fenton reactions and Haber-Weiss reactions, contributing to ROS generation. Under physiological conditions, the cells maintain a balance between ROS generation and antioxidant defenses. Any disturbances in this balance would lead to oxidative stress, resulting in damage to biomolecules and disruption of signaling pathways that implicate in different cell death mechanism such as apoptosis and ferroptosis. From the inflammation perspectives, copper can activate the nucleotide-binding oligomerization domain, leucine-rich repeat and pyrin domain-containing protein 3 (NLRP3) inflammasome pathway, resulting in pyroptosis. Taken together, it is possible to establish an intricate linkage between cuproptosis with apoptosis, ferroptosis and pyroptosis through ROS-induced signaling pathways and cellular events. Which may trigger a series of cell death events, thereby overcoming the cell death resistance of cancer cells

Cancer cells exhibit a much higher demand for copper due to their highly active metabolism as compared to normal cells [92]. Significantly elevated levels of copper in the serum of cancer patients compared to normal subjects have been observed in a variety of malignancies [9–11]. It is well-known that high levels of copper concentrated in cancer cells may act on different signaling pathways that are essential in cell proliferation, angiogenesis, and metastasis, contributing to tumorigenesis and tumor growth. Paradoxically, literature has documented that increased levels of copper in cancer cells can also exert anticancer effects [93]. Therefore, there is a growing interest in exploiting copper-based approaches in the field of cancer therapy. However, understanding the regulatory mechanisms of cancer process by disrupting copper homeostasis remains challenging. It is widely accepted that the anticancer activities of copper mainly involve the induction of oxidative stress by participating in Fenton and Waber-Heiss reactions to produce OH^•^, which in turn result in cellular damage and eventually cell death [94]. Moreover, the copper-based reactions can operate in a broader pH range at a more rapid rate as compared to other transition metals such as iron, offering considerable advantages for performance [95]. It should be noted that cancer cells can escape from regulated cell death pathways, which is an important hallmark of cancer, posing one of the significant challenges in cancer treatment [74]. The recent discovery of cuproptosis has provided a novel target to overcome the cell death resistance of cancer cells, further exemplifying the potential of copper in cancer therapy. Cuproptosis is a novel type of regulated cell death that is dependent on copper accumulation and primarily occurs in the cells relying on OXPHOS as the major metabolic pathway for energy production. Copper directly binds with the lipoylated proteins of the TCA cycle, leading to the aggregation of these lipoylated proteins and disruption of the iron-sulfur cluster proteins and consequently proteotoxic stress [3]. As mentioned above, copper can generate an excess of OH^•^ during the switching between Cu + and Cu2 + states, leading to oxidative stress that underlines different known forms of regulated cell death. Considering the fact that intracellular accumulation of surplus copper exceeding the threshold would lead to cuproptosis, a process associated with ROS production and oxidative stress, this work pays attention to the crosstalk between cuproptosis with oxidative stress-associated regulated cell death mechanisms in order to provide new targets and new ideas for targeted cancer therapy (Fig. 4).

Apoptosis is a major type of regulated cell death that has been long implicated in cancer treatment [71]. Many studies have revealed that excess copper can produce toxic levels of ROS that cause damage to biomolecules and induce apoptosis. An in vitro study reported that copper sulfate significantly enhanced mitochondrial ROS levels, triggering oxidative stress and mitochondrial-mediated apoptosis pathway in chicken hepatocytes [96]. Another investigation using male rat liver and BRL-3 A cell models indicated that exposure to copper oxide nanoparticles led to ROS-induced oxidative stress that in turn activated endoplasmic reticulum stress, resulting in apoptosis [97]. Excess copper can also induce apoptosis through death receptor signaling pathways. The combination of disulfiram (DSF) and Cu2 + was found to induce ROS production, triggering the proapoptotic-related c-Jun N-terminal kinase (JNK) and p38 mitogen-activated protein kinase (MAPK) pathways while inhibiting the anti-apoptotic nuclear factor kappa B (NF- κB) pathway, ultimately resulting in apoptosis of breast cancer cells [98]. It is worth mentioning that ROS can compromise the synthesis of iron-sulfur clusters, which play a role in a variety of crucial enzymes, some of which are important for apoptotic signaling pathways [99]. Moreover, mitochondrial stress can lead to a significant loss of mitochondrial membrane potential, promoting apoptosis [100]. Recently, copper has been found to interact with the tumor suppressor p53 that regulates cell cycle and apoptosis. Exposure to copper was found to cause the activation of p53, leading to the execution of apoptosis in epithelial breast cancer cells [101]. It is well-documented that cancer cells prefer glycolysis to OXPHOS for generating intermediate metabolites and energy, thereby inhibiting cuproptosis. Interestingly, p53 as a crucial metabolic regulator can inhibit glycolysis and switch the metabolic pathway into OXPHOS in cancer cells, facilitating cuproptosis. Moreover, p53 is known to regulate the biogenesis of iron-sulfur clusters and the copper chelator, namely GSH, further emphasizing the role of this tumor suppressor as an intermediate node between apoptosis and cuproptosis [102].

Another pivotal form of regulated cell death that has been documented to link to cuproptosis is ferroptosis, which is an iron-dependent cell death induced by iron accumulation and lipid peroxidation [103]. It was indicated that ferroptosis inducers promoted copper ionophore-induced cell death in primary liver cancer cells, suggesting that the agents can trigger not only ferroptosis but also cuproptosis. The mechanisms were ascertained to the upregulation of protein lipoylation by inhibiting mitochondrial matrix-related proteases mediated FDX1 degradation and reducing intracellular GSH synthesis [104]. While iron is considered as the primary inducer of ferroptosis, several studies have demonstrated the ability of copper to induce ferroptosis. It was found that treatment with copper ionophores led to copper-dependent ferroptosis in colorectal cancer cells via ATP7A degradation [105]. Furthermore, it was determined that disulfiram/copper elicited antitumor activities against both nasopharyngeal cancer cells and cancer-associated fibroblasts through ROS/MAPK and p53-mediated ferroptosis pathways [106]. Another study reported that disulfiram/copper led to impaired mitochondrial homeostasis, elevated free iron pool, and increased lipid peroxidation, eventually resulting in ferroptosis in hepatocellular carcinoma cells [107]. Mechanistically, excess copper can induce ROS generation and oxidative stress that further leads to lipid peroxidation damage to cell membrane, which is a prerequisite for ferroptosis [108]. Moreover, as a major source of intracellular ROS, mitochondria may play a crucial role in ferroptosis. In fact, it was demonstrated that mitochondria were essential in cysteine deprivation-induced ferroptosis but not in that induced by inhibiting glutathione peroxidase-4 (GPX4), the most downstream component of ferroptosis pathway. Mechanistically, mitochondrial TCA cycle and ETC could promote cysteine deprivation-induced ferroptosis by inducing mitochondrial membrane potential hyperpolarization and lipid peroxide accumulation. Importantly, loss of function of fumarate hydratase, a tumor suppressor and TCA cycle component, inhibited cysteine deprivation-induced ferroptosis [109]. These findings propose the crucial role of mitochondria in ferroptosis and implicate the mitochondrial TCA cycle as a converging node for ferroptosis and cuproptosis. In addition to mitochondrial metabolism, a recent review has suggested that GSH may serve as an additional common hub for ferroptosis and cuproptosis. On the one hand, GSH functions as an antioxidant that prevents lipid peroxidation in ferroptosis. On the other hand, GSH acts as a copper chaperone that binds copper to attenuate the aggregation of lipoylated proteins in cuproptosis. GSH has been shown to exert inhibitory effects on both ferroptosis and cuproptosis, indicating a co-regulatory crosstalk between these processes mediated by GSH [110].

From a standpoint of inflammation, the activation of the nucleotide-binding oligomerization domain, leucine-rich repeat and pyrin domain-containing protein 3 (NLRP3) inflammasome pathway has proposed the possibility of cuproptosis to interact with pyroptosis, which is an inflammatory form of lytic programmed cell death. Many studies have demonstrated that copper exposure can activate NLRP3 inflammasomes, resulting in pyroptosis. An in vivo study reported that the relationship between intracellular copper and NLRP3 inflammasome activation, and copper depletion significantly inhibited classical NLRP3-mediated pyroptosis in SOD1-deficient mice [111]. Treatment of murine macrophages with copper oxide nanoparticles led to increased levels of NLRP3, caspase-1, and interleukin-1β (IL-1β), along with increased release of IL-1β, ultimately leading to NLRP3-dependent pyroptosis [112]. Similar results were found in primary microglia treated with copper dichloride, which exhibited elevated levels of proinflammatory elements including NLRP3, cleaved caspase-1, ASC, and IL-1β [113]. Intriguingly, a recent study found that self-destructive and multienzymatically active copper-based nanoparticles can affect the antioxidant defense mechanism of cancer cells by inhibiting the nuclear factor-erythroid 2-related factor 2-quinone oxidoreductase 1 signaling pathway. Furthermore, the material was able to activate not only NLRP3-mediated pyroptosis but also cuproptosis, both of which resulted in immunosuppressive tumor microenvironment remodeling, increased infiltration of immune cells, and profound systemic immunity [114]. These findings therefore highlight the potential interplay between cuproptosis and pyroptosis, particularly via the canonical pathway.

## Therapeutic implications of copper-induced oxidative stress for targeting cuproptosis and inducing cancer cell death

Recent progress in the study of copper-dependent signaling offers translational prospects for the chemistry and biology of copper into clinical practice that leverage disease vulnerabilities to such metal nutrient. This proof-of-concept is particularly relevant in cancer as cancer cells require much more copper for their highly active metabolism. However, excessive copper intake can induce cell death through cytotoxicity resulted from increased mitochondrial-dependent energy metabolism and ROS accumulation in a process termed cuproptosis [115]. Herein, this review suggests some potential strategies to harness these mechanisms for enhancing the effectiveness of cancer treatment. By strategically modulating copper levels, enhancing ROS production, and employing combination treatments, it is possible to exploit the distinctive vulnerabilities that are inherent to cancer cells.

A potential strategy that can selectively induce cuproptosis in cancer cells is to modulate copper levels within the tumor microenvironment since cancer cells have a higher demand for this metal nutrient compared to normal cells. A variety of copper-binding compounds termed copper ionophores have been found to exert anticancer activities by transporting this element into cells and increasing its intracellular bioavailability, triggering cell death. Among different classes of copper ionophores, ES has exhibited acceptable safety and therapeutic efficiency in the clinical practice. A phase I clinical trial in patients with refractory solid tumors showed that the ES/paclitaxel combination was well tolerated with a toxicity profile similar to single-agent paclitaxel [116]. In a multicenter phase II, randomized, controlled, double-blinded trial for stage IV metastatic melanoma, treatment with ES plus paclitaxel showed a doubling of progression-free survival time compared with treatment with paclitaxel alone, with an acceptable toxicity profile and encouraging overall survival [117]. A randomized, double-blinded, controlled phase III study in patients with stage IV chemotherapy-naïve melanoma revealed a significant improvement in progression-free survival for ES in combination with paclitaxel versus paclitaxel alone in the subgroup of patients with normal baseline lactate dehydrogenase levels [118]. However, the mechanism underlying the anticancer activity of ES has not been fully understood. ES was initially recognized as an inducer of oxidative stress [119], but recently it has also been demonstrated to suppress cancer by inducing cuproptosis [3]. These findings imply that the use of copper-binding compounds to sensitize tumor cells to copper-induced cell death may be a part of clinical regimens. It is worth noting that the prolonged use of such medications can disturb the homeostasis of trace elements, resulting in adverse effects in patients undergoing the treatment. Although copper-binding compounds may have selective effects on cancer cells, the next generation of these agents should exploit targeting molecules that can be recognized only by specific receptors exclusively found in cancer cells for safer applications.

Another potential strategy that can exploit copper-induced cell death is induction of ROS generation. The proof-of-concept is that agents disrupting the cellular redox balance through increased intracellular ROS levels can push cells towards oxidative stress and subsequent cell death. Active compounds have both a metal-binding domain and domains capable of mediating passage across the plasma membrane. A well-recognized metal-binding domain is the thiol (–SH) group, which can be found in various molecules [120]. N-acetylcysteine (NAC) is de-acetylated to cysteine either on the cell surface or inside of the cell [121], thereby promoting GSH formation when intracellular cysteine is limited and enhancing the antioxidant activity. NAC has been employed for many years as an antioxidant in biomedical research and clinical practice [122, 123]. However, since antioxidants may act as pro-oxidants under certain circumstances, it is not surprising that NAC has also been reported to promote DNA damage due to, in part, increased ROS generation [124–126]. Copper in combination with NAC has shown promising results in producing ROS and inducing cell death. It was demonstrated that the combination led to significant ROS generation, enhancing oxidative stress and selectively inducing cell death in human ovarian cancer cells [127]. Consistently, a recent study reported that the combinations of copper-containing nanoparticles and organic complexes with NAC were remarkably cytotoxic for a panel of human cancer cell lines, including the variants with the molecular determinants of altered drug resistance. Importantly, every single component yielded little-to-no activity, whereas together, the effects were significant. Cell death was observed for a variety of cell lines regardless of the tissue origin, which was accompanied by rapid ROS generation followed by plasma membrane perturbations but not caspase activation or PARP cleavage, suggesting a mode of ROS-induced non-apoptotic cell death [128]. These findings suggest that copper/NAC interactions could be leveraged as a part of regimens to induce oxidative stress and trigger copper-induced cell death in cancer therapy.

The emerging strategy of integrating the known cell death with cuproptosis may hold a great potential in clinical oncology. This novel strategy seeks to enhance the efficacy of cancer treatment by capitalizing on the unique vulnerabilities of cancer cells. Moreover, repurposing existing medications to uncover their roles associated with copper-induced cell death offer a promise for dealing with cancer. For instance, the reciprocity between regulators of ferroptosis and cuproptosis defined the tumor microenvironment in hepatocellular carcinoma and lung adenocarcinoma, determining prognosis and sensitivity for chemotherapeutics [129, 130]. It was disclosed that sorafenib and erastina as inducers of iron-dependent cell death were able to inhibit intracellular GSH synthesis and FDX1 degradation, which in turn led to cuproptosis by enhancing the oligomerization of lipoylated proteins [104]. In contrast, a recent study revealed that excessive accumulation of copper induced by the ES copper ionophore could suppress the growth and proliferation of colorectal cancer cells through ferroptosis. ES also promoted ROS production, resulting in the enhancement of oxidative stress and the subsequent reduction in the expression of copper- transporting ATP7A, induction of ferroptosis, and SLC7A11 degradation in colorectal cancer cells [105]. Thus, ES may be considered as a co-regulator of both the form of cell death. Another copper ionophore, namely DSF, has also exhibited promising antineoplastic potentials with copper. DSF was reported to induce ferroptosis by increasing free iron through mitochondrial disruption along with attenuation in the proliferation and migration of liver and nasopharyngeal cancer cells [106, 107]. In mouse models of pancreatic cancer, copper overabundance through ionophores also enhanced the ferroptosis mechanism [131]. In order to develop more potent anticancer therapeutics, future research may focus on copper-targeted interventions in combination with other treatments, contributing to innovative solutions and improving clinical outcomes. However, it is crucial to establish a proper balance between copper metabolism and therapeutic interventions in order to elicit desirable performance while minimizing the toxicities and side effects.

## Conclusion

The discovery of cuproptosis offers a novel approach for cancer therapy by exploiting the pathophysiological role of copper. The present review comprehensively outlines the predominant mechanisms of cuproptosis for therapeutic purposes, which are intricately linked to apoptosis, ferroptosis and pyroptosis through ROS-induced signaling pathways and cellular events. Such crosstalk between different forms of regulated cell death may trigger a series of cell death events, thereby overcoming the cell death resistance of cancer cells. As the field of cuproptosis is nascent in many ways, further studies are required to fully elucidate the mechanisms connecting modes of cell death. Moreover, determining the precise range of copper levels that cause selective cytotoxicity to cancer cells for the translation of personalized copper-based strategies for targeting specific cell death pathways is of importance.

## Data Availability

No datasets were generated or analysed during the current study.
